# Complete chloroplast genome data of *Plumbago indica* L. a prominent medicinal plant

**DOI:** 10.1016/j.dib.2025.112369

**Published:** 2025-12-10

**Authors:** Jiaqiang Zhang, Songlin Wang, Shizhen Wang, Yuan Zhou, Bowei He, Juan Zhang

**Affiliations:** a Zhejiang Institute of Landscape Plants and Flowers, Zhejiang Academy of Agricultural Sciences, Hangzhou 311251, China; b Agricultural Technology Extension Center of Zhejiang Province, Hangzhou 310020, China

**Keywords:** Chloroplast genome, *Plumbago indica*, Phylogenetic tree

## Abstract

*Plumbago indica* L., a herbaceous plant with significant medicinal value, belongs to the Plumbaginaceae family. In this study, we assembled and report the complete chloroplast genome of *Plumbago indica*. The chloroplast genome of *Plumbago indica* was 169,025 bp in size, consisting of a large single-copy region (91,683 bp), a small single-copy region (SSC) (13,420 bp), and a pair of inverted repeat regions (IRs) (31,961 bp). It contains 129 genes, including 84 protein-coding genes, 37 tRNA genes, and 8 rRNA genes. Phylogenetic analysis showed that *Plumbago indica* forms a close relationship with *Plumbago auriculata* and *Plumbago zeylanica* within the Plumbaginaceae family. This was the first complete report of the chloroplast genome of *Plumbago indica* providing an opportunity to explore genetic diversity and contributing to species identification and conservation efforts.

Specifications Table

**Table:** 

Subject	Biology
Specific subject area	Omics: Chloroplast Genomics
Type of data	Tables, Figures, Raw
Data collection	Fresh leaves were collected, and total genomic DNA was extracted. Chloroplast genome was de novo assembled using NOVOPlasty. Annotation was performed using GeSeq with manual curation. Genome visualized using CPGAVAS2. SSR analysis using MISA. Phylogenetic analysis using MAFFT, and MEGA with Maximum likelihood (ML) and Neighbor-Joining(NJ).
Data source location	City: Hangzhou City, Zhejiang Province Country: China Latitude and longitude:120°2338,30°0761. Voucher Specimen: Deposited at Zhejiang Institute of Landscape Plants and Flowers Herbarium under voucher number ZHD2022090602 (Curator: Qiang Chang, Email: 394409936@qq.com).
Data accessibility	Repository name: NCBI (National Center for Biotechnology Information) BioProject: PRJNA1303398 (https://www.ncbi.nlm.nih.gov/bioproject/PRJNA1303398/) GenBank Accession Number: PX136589 (https://www.ncbi.nlm.nih.gov/nuccore/PX136589.1/)

## 1 Value of the Data

• DNA barcoding: Based on the full-length chloroplast genome DNA barcoding, *Plumbago indica* can be distinguished from their close relatives, resolving the confusion in the medicinal market.
• Intraspecific relationships: By comparing the chloroplast genomes of species within the Plumbaginaceae family, it was confirmed that *Plumbago indica* forms a monophyletic group with its same genus species, supporting its taxonomic status.
• Genetic resource conservation: The chloroplast genome data provide a molecular basis for the evaluation of the germplasm resources, population genetic diversity analysis, and the formulation of conservation strategies of *Plumbago indica*.

## 2 Background

*Plumbago indica* is a small shrub or perennial plant that grows in warm tropical climates. It has a long history of application in traditional herbal medicine and Chinese medicine, especially in Ayurvedic medicine, where it was highly regarded for its efficacy in promoting digestion, anti-inflammatory, deworming, expectorant, diaphoretic, diuretic, and laxative effects. Its roots and leaves to treat various diseases, such as gastrointestinal disorders, skin diseases, respiratory system diseases, and rheumatic diseases [1,2]. It contains a variety of plant chemical components and was prolific in naphthoquinone compounds. Among them, Plumbagin (5-Hydroxy-2-methyl-1, 4-naphtho-quinone) was the therapeutic active component in the roots of *Plumbago indica*. Plumbagin has shown potential utility in inflammation, free radical reduction, bacterial control and cancer [3,4].

Due to increased demand, reduced wild population, low germination rate, and slow root growth of *Plumbago indica*, situations of species substitution and/or adulteration occurred in the herbal market [5]. Regarding the adulteration issue in the *Plumbago indica* market, the DNA barcode technology in the ITS2 region has been identified as an effective method to distinguish orther Plumbago species [6]. A recent study applied the MassArray iPLEX assay for differentiating Plumbago species [7]. However, due to the large number of SNPs and SSR loci among plants, it was necessary to develop more differential loci to distinguish the authenticity and origin of *Plumbago indica*.

The chloroplast genome follows a maternal inheritance pattern and was transferred to offspring by the mother in most angiosperms. The sequence conservation of the chloroplast genome was relatively high, but there are sufficient variable sites (SNP, InDel), which are ideal molecular markers for precise species identification (DNA barcoding) and reconstruction of plant phylogenetic relationships. Although the chloroplast genomes of closely related species such as *Plumbago zeylanica* and *Plumbago auriculata* have been sequence and analysed [8,9], the complete chloroplast genome sequence data of *Plumbago indica* has not been officially reported according to the existing public literature databases (NCBI GenBank) and provided literature materials. Therefore, decoding the chloroplast genome of *Plumbago indica* and conducting phylogenetic analysis of related species will help determine the phylogenetic relationship of *Plumbago indica* in the Plumbaginaceae family and provide assurance for drug safety.

## 3 Data Description

**Fig. 1:**
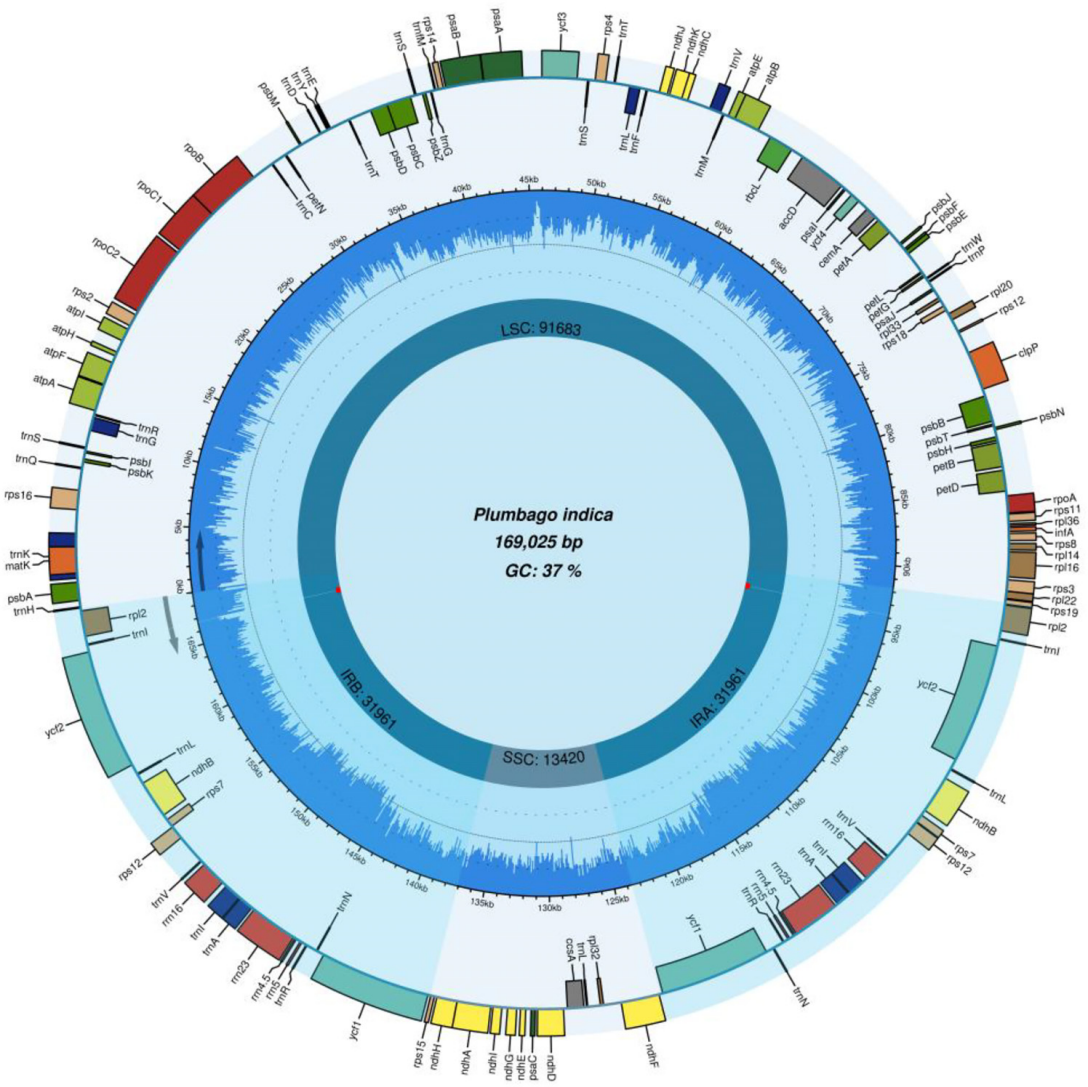
The chloroplast genome map of *Plumbago indica.* The quadripartite structure was shown; LSC, SSC, IRA and IRB are labelled according to defined boundaries.

The total length of the chloroplast genome of *Plumbago indica* was 169,025 bp, with a GC content of 37.22 %. The chloroplast genome was divided into four main regions: LSC (large single-copy region), length of 91,683 bp. SSC (small single-copy region), length of 13,420 bp. IRa and IRb (reverse repeat regions), each region was 31,961 bp in length. It contains 129 genes, including 84 protein-coding genes, 37 tRNA genes and 8 rRNA genes (Fig. 1).

Based on their functions, these were classified into 4 major categories (Table 1), including genes related to photosynthesis (43), genes related to self-replication (65), other genes (6), and genes of unknown function (4). Among these genes, 17 genes (ndhB, rpl2, rps12, rps7, rrn16S, rrn23S, rrn4.5S, rrn5S, trnA-UGC, trnI-CAU, trnI-GAU, trnL-CAA, trnN-GUU, trnR-ACG, trnV-GAC, ycf1, ycf2) exhibit duplication. Additionally, 15 genes (ndhA, ndhB, petB, petD, atpF, rpl16, rps16, rpoC1, trnA-UGC, trnG-UCC, trnI-GAU, trnK-UUU, trnL-UAA, trnV-UAC, accD) have turned out to contain one intron. It was noteworthy that the three specific genes, ycf3, clpP, and rps12, each have two introns of their own.

**Table 1:** List of genes found in four chloroplast genomes of *Plumbago indica.*

Category	Gene group	Gene name
Photosynthesis	Subunits of photosystem I	psaA,psaB,psaC,psaI,psaJ
	Subunits of photosystem II	psbA,psbB,psbC,psbD,psbE,psbF,psbH,psbI,psbJ,psbK,psbM,psbN,psbT,psbZ
	Subunits of NADH dehydrogenase	ndhA*,ndhB*(2),ndhC,ndhD,ndhE,ndhF,ndhG,ndhH,ndhI,ndhJ,ndhK
	Subunits of cytochrome b/f complex	petA,petB*,petD*,petG,petL,petN
	Subunits of ATP synthase	atpA,atpB,atpE,atpF*,atpH,atpI
	Large subunit of rubisco	rbcL
	Subunits photochlorophyllide reductase	-
Self-replication	Proteins of large ribosomal subunit	rpl14,rpl16*,rpl2*(2),rpl20,rpl22,rpl32,rpl33,rpl36
	Proteins of small ribosomal subunit	rps11,rps12**(2),rps14,rps15,rps16*,rps18,rps19,rps2,rps3,rps4,rps7(2),rps8
	Subunits of RNA polymerase	rpoA,rpoB,rpoC1*,rpoC2
	Ribosomal RNAs	rrn16S(2),rrn23S(2),rrn4.5S(2),rrn5S(2)
	Transfer RNAs	trnA-UGC*(2),trnC-GCA,trnD-GUC,trnE-UUC,trnF-GAA,trnG-GCC,trnG-UCC*,trnH-GUG,trnI-CAU(2),trnI-GAU*(2),trnK-UUU*,trnL-CAA(2),trnL-UAA*,trnL-UAG,trnM-CAU,trnN-GUU(2),trnP-UGG,trnQ-UUG,trnR-ACG(2),trnR-UCU,trnS-GCU,trnS-GGA,trnS-UGA,trnT-GGU,trnT-UGU,trnV-GAC(2),trnV-UAC*,trnW-CCA,trnY-GUA,trnfM-CAU
Other genes	Maturase	matK
	Protease	clpP**
	Envelope membrane protein	cemA
	Acetyl-CoA carboxylase	accD*
	c-type cytochrome synthesis gene	ccsA
	Translation initiation factor	infA
	other	-
Genes of unknown function	Conserved hypothetical chloroplast ORF	ycf1(2),ycf2(2),ycf3**,ycf4

Notes: Gene*: Gene with one introns; Gene**: Gene with two introns; #Gene: Pseudo gene; Gene(2): Number of copies of multi-copy genes.

**Fig. 2:**
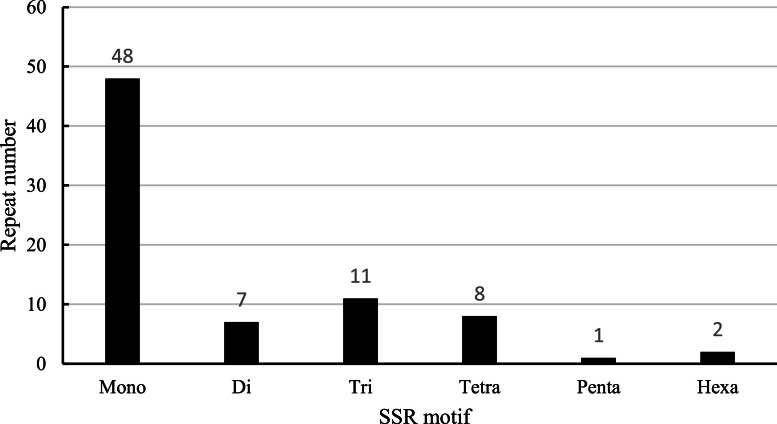
Simple sequence repeat (SSR) analysis in *Plumbago indica* chloroplast genomes.

We identified a total of 77 simple sequence repeats (SSR) use the microsatellite technology. After using the relevant detection methods, six types of SSR were discovered, 48 single nucleotides, 2 dinucleotides, 11 trinucleotides, 8 tetranucleotides, 1 pentanucleotide and 2 hexanucleotides (Fig. 2). The proportion of single nucleotides was the highest.

**Fig. 3:**
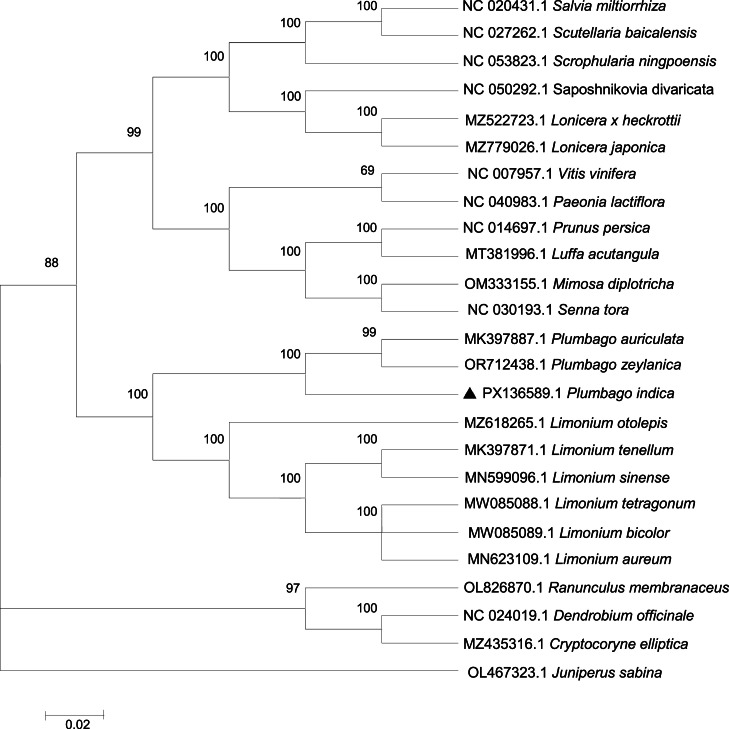
The phylogenetic tree of 25 species constructed based on the maximum likelihood (ML) method of chloroplast genome sequence. ▲denotes species analysed in this study.

**Fig. 4:**
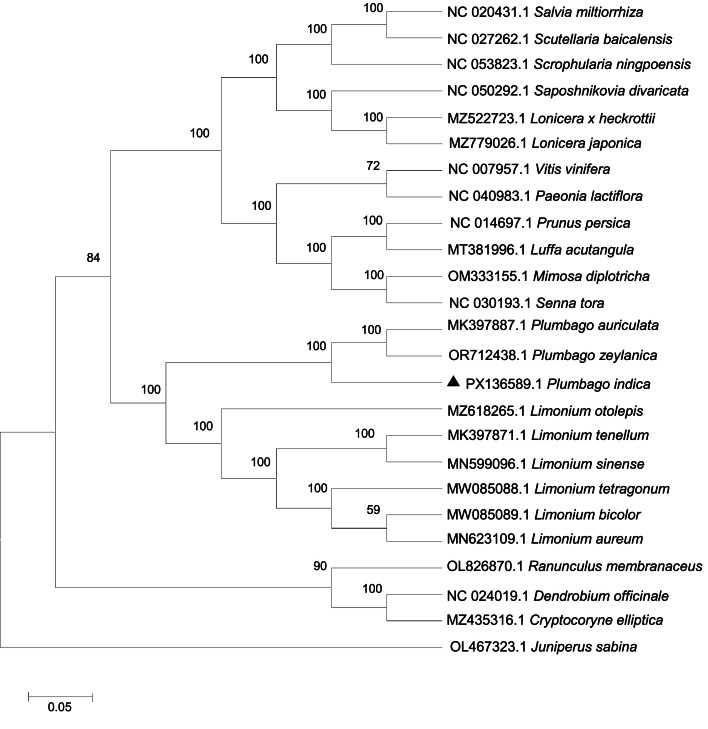
Phylogenetic tree of 25 species constructed based on Neighbor-Joining (NJ) method of chloroplast genome sequence. ▲denotes species analysed in this study.

This study constructed a phylogenetic tree centered on *Plumbago indica* using the maximum likelihood method, and its topological structure (Fig. 3). This phylogenetic tree clearly demonstrates the branching relationships between *Plumbago indica* and other related species. In the phylogenetic tree, *Plumbago indica* was located on an independent branch of the Plumbaginaceae family, forming a sister group relationship with other species in the same family such as *Plumbago auriculata* and *Plumbago zeylanica*. Moreover, the phylogenetic tree also reveals the divergence nodes between *Plumbago indica* and Limonium species. To further verify the accuracy of the topological structure, we conducted a comparative analysis using Neighbor-Joining method (Fig. 4), and the results showed that the main branch relationships remained consistent, further supporting the reliability of the phylogenetic tree.

## 4 Experimental Design, Materials and Methods

### 4.1 Plant samples

Fresh specimens of *Plumbago indica* were collected from the Zhejiang Institute of Landscape Plants and Flowers in Xiaoshan District, Hangzhou City, Zhejiang Province (coordinates: 120°23′38″ E, 30°07′61″N). The plant materials are stored in the specimen room of this institute at 394,409,936@qq.com (registration number: ZHD2022090602). The collection of the specimens fully consistent with the regulations for the protection of wild animals in China. The complete genomic DNA was extracted from the plant samples using the improved CTAB technique [10].

### 4.2 Sequencing and sequence analyses

The genome sequence was conducted using the Illumina Hiseq 2500 platform (Illumina, USA), and *Plumbago auriculata* (NCBI GenBank: MH286308) was used as a reference sequence. The sequence data quality was evaluated with FastQC v0.11.4 package [11] using of default parameters. The assembled chloroplast genome was annotated using CPGAVAS2 software [12]. The circular genome map was brought with OrganellarGenomeDRAW (OGDRAW) v1.3.1 [13].

### 4.3 Simple sequence repeat (SSR) analysis

The simple sequence repeat (SSR) analysis of the chloroplast genome of *Plumbago indica* was conducted using MISA (https://webblast.ipk-gatersleben.de/misa/) [14]. The parameters set by this platform were as follows:‘10' for mono-, ‘5' for di-, ‘4' for tri-, ‘3' for tetra-, ‘3' forpenta- and ‘3' for hexa-nucleotide motifs.

### 4.4 Phylogenetic tree analysis

To determine the phylogenetic position of *Plumbago indica*, complete chloroplast genome sequences of 25 other species were downloaded from NCBI GenBank (Accession numbers provided in Fig. 3). The sequences were aligned using MAFFT v7 [15]. Maximum likelihood (ML) using the Tamura-Nei model and 1000 bootstrap replications and Neighbor-Joining (NJ) with 1000 bootstrap replications phylogenetic tree was constructed using MEGA 6.0 [16], with *Juniperus sabina* (OL467323) as the outgroup.

## Limitations

None.

## Ethics Statement

All authors have read and follow the ethical requirements for publication in Data in Brief and confirming that the current work does not involve human subjects, animal experiments, or any data collected from social media platforms.

## CRediT Author Statement

**Jiaqiang Zhang:** Data curation, Formal analysis, Writing–original draft. **Songlin Wang:** Investigation. **Shizhen Wang:** Visualization Methodology. **Yuan Zhou:** Resources, Validation. **Bowei He:** Supervision, Project administration, Writing–review & editing. **Juan Zhang:** Conceptualization, Funding acquisition, Writing–review & editing.

## Data Availability

Original dataPlumbago indica chloroplast, complete genome.

## References

[1] A.Abdu A.Prakash, R. Kondal S.Sharma, Bhagat M., Pal R., Singh H., Singh B., Kaur S. (2024). Comprehensive review on *Plumbago indica*: traditional, pharmacological insights and conservation strategie. J. Appl. Pharm. Sci..

[2] Priyanjani H.A.S.A, Senarath R.M.U.S., Senarath W.T.P.S.K, Munasinghe M.L.A.M.S. (2021). propagation, phytochemistry and pharmacology of *Plumbago indica* - a review. J. Pharm. Res. Int..

[3] Hemant B., Dinesh K., Dharmendra A. (2022). Phytochemistry and pharmacological activities of *Plumbago indica* L.: an overview. Int. J. Pharm. Investig..

[4] Khatun A. (2023). *Plumbago indica* L.: a review of its medicinal uses, phytochemistry, pharmacology, and toxicology. Int. J. Herb. Med..

[5] Pandey D.K., Katoch K., Das T., Majumder M., Dhama K., Mane A.B., Gopalakrishnan A.V., Dey A. (2023). Approaches for in vitro propagation and production of plumbagin in Plumbago spp. Appl. Microbiol. Biotechnol..

[6] Sharma B., Dhiman C., Hasan G.M., Shamsi A., Hassan M.I. (2024). Pharmacological features and therapeutic implications of plumbagin in cancer and metabolic disorders: a narrative review. Nutrients.

[7] Thongkhao K., Intharuksa A., Phrutivorapongkul A. (2025). Unveiling adulteration in herbal markets: massARRAY iPLEX Assay for accurate identification of *plumbago indica* L. Int. J. Mol. Sci..

[8] Zhou H., Zhang H. (2024). The complete chloroplast genome of the medicinally important plant *Plumbago zeylanica* L. (plumbaginaceae) and phylogenetic analysis. Mitochondrial DNA B Resour..

[9] Darshetkar A.M., Maurya S., Lee C., Bazarragchaa B., Batdelger G., Janchiv A., Jeong E.J., Choi S., Choudhary R.K., Y.Kim S. (2021). Plastome analysis unveils inverted repeat (IR) expansion and positive selection in sea lavenders (Limonium, Plumbaginaceae, Limonioideae, Limonieae). PhytoKeys.

[10] Allen G.C., Flores-Vergara M.A., Krasynanski S., Kumar S., Thompson W.F. (2016). A modified protocol for rapid DNA isolation from plant tissues using cetyltrimethylammonium bromide. Nat. Protoc..

[11] De B.G.S., Smith A.D. (2019). Falco: high-speed FastQC emulation for quality control of sequencing data. F1000 Res..

[12] Shi L., Chen H., Jiang M., Wang L., Wu X., Huang L., Liu C. (2019). CPGAVAS2, an integrated plastome sequence annotator and analyzer. Nucleic Acids Res..

[13] Greiner S., Lehwark P., Bock R. (2019). OrganellarGenomeDRAW (OGDRAW) version 1.3.1: expanded toolkit for the graphical visualization of organellar genomes. Nucleic Acids Res..

[14] Beier S., Thiel T., Münch T., Scholz U., Mascher M. (2017). MISA-web: a web server for microsatellite prediction. Bioinformatics.

[15] Katoh K., Standley D.M. (2013). MAFFT multiple sequence alignment software version 7: improvements in performance and usability. Mol. Biol. Evol..

[16] Kumar S., Stecher G., Li M., Knyaz C., Tamura K. (2018). MEGA X: molecular phylogenetic genetics analysis across computing platforms. Mol. Biol. Evol..

